# *HER2/CEP17 *ratio predicts residual cancer burden after neoadjuvant dual HER2 blockade: real-world data in patients with primary HER2-amplified breast cancer

**DOI:** 10.1007/s00428-025-04203-5

**Published:** 2025-08-05

**Authors:** Myriam Stolz, Alex Farr, Kristina A. Tendl-Schulz, Florian Frommlet, Helga Reckendorfer, Oskar Koperek, Barbara Neudert, Maximilian Marhold, Ulrike Heber, Ruth Exner, Christian F. Singer, Rupert Bartsch, Zsuzsanna Bago-Horvath

**Affiliations:** 1 Department of Obstetrics and Gynecology, Comprehensive Cancer Center, Medical University of Vienna, Vienna, Austria; 2 Department of Pathology, Comprehensive Cancer Center, Medical University of Vienna, Waehringer Guertel 18–20, 1090 Vienna, Austria; 3 Institute of Medical Statistics, Informatics and Intelligent Systems (CeMSIIS), Medical University of Vienna, Vienna, Austria; 4 Institute of Clinical Pathology, Cytology and Microbiology Ltd. Vienna, Dr. Kosak Und Partner - Fachärzte Für Pathologie GmbH, Vienna, Austria; 5 Laboratory Kaserer, Koperek and Beer Ltd., Vienna, Austria; 6 Department of Medicine I, Division of Oncology, Comprehensive Cancer Center, Medical University of Vienna, Vienna, Austria; 7 Department of General and Visceral Surgery, Division of Oncology, Comprehensive Cancer Center, Medical University of Vienna, Vienna, Austria

**Keywords:** Breast cancer, Neoadjuvant therapy, HER2/CEP17 ratio, Residual cancer burden

## Abstract

Novel human epidermal growth factor receptor 2 (HER2)-directed therapies have significantly improved outcomes for patients with HER2-positive early-stage breast cancer. Our study assessed the impact of *HER2/chromosome enumeration probe 17* (*CEP17*) ratio on residual cancer burden (RCB) and long-term prognosis following neoadjuvant chemotherapy with dual HER2-targeted therapy using trastuzumab and pertuzumab to identify candidates for chemotherapy de-escalation. Our study included 169 patients with primary invasive HER2-positive breast cancer who received neoadjuvant chemotherapy with trastuzumab and pertuzumab at the Medical University of Vienna from 2014 to 2020. *HER2 (ERBB2)* gene copy number and *HER2/CEP17* ratio were assessed by in situ hybridization. RCB and pathologic complete remission (pCR) served as primary and secondary endpoints, respectively. Univariate and multivariate logistic regression models were applied to analyze associations between outcomes and predictor variables, focusing on the predictive role of *HER2/CEP17* ratio, with cutoff values estimated. Progression-free survival (PFS) and overall survival (OS) were estimated by the Kaplan–Meier method. *HER2/CEP17* ratio was significantly associated with response to dual-targeted neoadjuvant chemotherapy in primary HER2-positive breast cancer. Optimal *HER2/CEP17* cutoff for predicting RCB 0/I was identified at 5.19. *HER2/CEP17* ratio was also significantly associated with PFS as a continuous predictor. Multivariate analysis showed that hormone receptor status and the presence of an in situ tumor component significantly influenced therapy response. HER2/CEP17 ratio predicts therapy response and therefore might aid patient stratification for therapy de-escalation with respect to dual neoadjuvant HER2 blockade. Further investigations are warranted to confirm its relevance with other HER2-targeted agents.

## Introduction

The advent of combined neoadjuvant chemo-immune therapy has revolutionized the treatment of *human epidermal growth factor receptor 2* (*HER2*)-amplified early-stage breast cancer, achieving pathologic complete response (pCR) rates of up to ≥ 50% [1]. Since then, HER2 testing guidelines and the definition of HER2 positivity have not been substantially re-evaluated with respect to novel HER2-targeted agents [2, 3]. At present, the predictive value of HER2 status regarding neoadjuvant therapy response at the individual patient level leaves room for improvement.

The NeoSphere and TRYPHAENA studies demonstrated that addition of the synergistically acting monoclonal antibody pertuzumab to trastuzumab-based regimens improved pCR rates [1, 4]. However, increased response rates to dual HER2 blockade were mostly limited to hormone receptor (HR)-negative non-luminal tumors. In these patients, significantly longer progression-free survival (PFS) rates in the 5-year analysis consequently suggest a carry-over effect for long-term survival [5]. Improved long-term outcomes have also been confirmed in previous meta-analyses [6, 7]. Consequently, neoadjuvant dual chemo-immunotherapy with trastuzumab and pertuzumab has become the standard of care for primary HER2-positive invasive breast cancer [8]. HER2 status is usually determined in core needle biopsy (CNB) specimens, as amplification status has been shown to remain stable during neoadjuvant therapy [9]. Nevertheless, a significant number of patients fail to respond adequately to dual HER2 blockade and require further post-neoadjuvant chemotherapy to prevent relapse [10]. In contrast, several patients achieve pCR after a trastuzumab-only or an antibody-only regime and therefore may be overtreated by the current standard [11, 12]. Therefore, reliable predictive factors are required to individually tailor HER2-targeted therapies to avoid over- and under-treatment and minimize adverse effects [13].

pCR is a valid predictor of long-term survival; however, residual cancer burden (RCB) may provide more accurate prognostic information using a continuous score without dichotomization [14–16]. RCB is an independent predictor of prognosis after neoadjuvant therapy and outperforms pCR in predicting long-term survival in all subtypes of breast cancer [17]. In fact, pCR demonstrates weak trial-level associations and—unlike RCB—may not be considered a surrogate parameter for PFS or overall survival (OS) [18]. Therefore, assessing response by RCB is likely to improve patient selection for de-escalation of post-neoadjuvant therapy concept without jeopardizing survival. Currently, pCR plays an important role in post-neoadjuvant therapy decisions only [19].

The level of *HER2* amplification (as defined by the *HER2* gene copy number and *HER2/*chromosome enumeration probe 17 ratio) has been demonstrated earlier to serve as a predictor of response to HER2-targeted treatments at the individual patient level [20–23]. Emerging gene expression-based assays have also confirmed the predictive role of *ERBB2* mRNA expression after neoadjuvant dual HER2 blockade in combination with chemotherapy [24].

The limitations of these earlier studies include the mostly small sample size and the inclusion of patients receiving both trastuzumab only as well as dual HER2 blockade [20, 22, 25–27]. One more important limitation in earlier analysis was the use of FISH instead of CISH, which compromises the exact quantification of clustered HER2 signals [26]. In addition, the primary endpoint of these investigations was generally pCR, instead of RCB and long-term outcome. Therefore, the exact correlation between *HER2/CEP17* ratio and PFS/OS remains to be clarified [16, 28, 29].

Our study analyzed whether *HER2/CEP17* ratio and *HER2* copy number can predict response, in terms of RCB, to current standard neoadjuvant dual HER2-targeted therapy and survival in a homogenous patient cohort receiving neoadjuvant chemotherapy plus trastuzumab and pertuzumab. We also aimed to investigate whether dichotomizing *HER2/CEP17* ratio at the previously suggested cutoff of 6 is useful in clinical practice or whether a better cutoff can be identified.

## Patients and methods

### Patients

Adult women with early-stage HER2-positive breast cancer who received neoadjuvant chemo-immunotherapy with dual HER2 inhibition using trastuzumab and pertuzumab at the Breast Center of the Medical University of Vienna between January 2014 and March 2020 were considered eligible. Treatment consisted of four cycles of epirubicin 90 mg/m^2^ in combination with cyclophosphamide 600 mg/m^2^ followed by four cycles of docetaxel 100 mg/m^2^ every 3 weeks with concomitant trastuzumab (8 mg/kg loading dose, then 6 mg/kg every 3 weeks) and pertuzumab (840 mg loading dose, then 420 mg every 3 weeks). All patients underwent tumor resection after neoadjuvant treatment. This study was approved by the Institutional Review Board of the Medical University of Vienna (protocol number: 1723/2019).

#### Pathology and *HER2/CEP17* in situ hybridization analysis

Histopathological tumor type was defined as described by the current World Health Organization classification of breast tumors [30]. Tumor grading was performed according to Elston and Ellis [31]. Tumor response was quantified by two expert pathologists using the RCB method [15, 28]. To reflect individual prognosis, RCB was dichotomized into two groups, RCB 0/I and RCB II/III, as customary in the existing literature [17].

HER2 status was assessed and reported according to the updated 2018 American Society of Clinical Oncology/College of American Pathologists (ASCO/CAP) guidelines [2, 32]. CNBs of all tumors were assessed by chromogenic in situ hybridization (CISH) using a Ventana HER2 Dual ISH DNA Probe cocktail, according to the manufacturer’s protocol. CISH analysis was preferred over fluorescence in situ hybridization (FISH), as by FISH, most cases presenting IHC score of 3 +, a cluster amplification is visible, making it almost impossible to count well single signals. By CISH, this hindrance was not present. Immunohistochemical stains and CISH slides were retrieved and independently re-evaluated for this study. Immunohistochemical results corresponded to the previous diagnostic evaluation in all cases, including 2 tumors with 3 + IHC stainings, in which subsequent CISH revealed no HER2 amplification. CISH was consensually evaluated by two experienced breast pathologists (KT, ZBH), and exact *HER2* gene copy numbers as well as *HER2/CEP17* ratios were determined. Upon CISH of review 2 + cases, no case had to be reassigned into a different diagnostic group according to current ASCO/USCAP categories (data not shown).

### Statistical analysis

For the descriptive analysis, categorical variables were summarized using absolute and relative frequencies. For metric variables, the median, minimum, and maximum values were used as summary statistics. Differences in *HER2/CEP17* ratios and *HER2* gene copy numbers between the groups for each outcome variable were assessed using the Wilcoxon rank-sum test or the Welch two sample *t*-test and were illustrated with grouped boxplots.

For RCB and pCR, logistic regression analyses were performed in an exploratory manner. Univariate models were computed for clinical and pathological predictor variables. To obtain multivariate logistic regression models for each outcome, all subset selections were performed using the Bayesian information criterion (BIC).

Progression-free survival (PFS) was defined as the time from the beginning of treatment until documented tumor progression or death, and overall survival (OS) was defined as the time from the beginning of treatment to death [33, 34]. The R package CatPredi (version 4.3, http://www.r-project.org/; R Development Core Team, Boston, MA, USA) was used to visualize the functional relationship between the log odds of the RCB/pCR and the predictors *HER2/CEP17* ratios and *HER2* gene copy numbers and to estimate potential optimal cutoff points when dichotomizing the predictor variables [35].

For recurrence and survival, Kaplan–Meier curves were plotted and stratified based on RCB and pCR, respectively. For PFS, the association with *HER2/CEP17* ratio was assessed using Cox regression, and log-rank tests were computed for group comparison at different cutoff values. Statistical analyses and figures were prepared using R version 4.3 (http://www.r-project.org/; R Development Core Team, Boston, MA, USA).

## Results

### Clinical characteristics

In total, 198 patients with HER2-positive breast cancer met the inclusion criteria. Of these, 29 (14.6%) were excluded because of missing or inconsistent data or disease progression during neoadjuvant therapy. Breast-conserving surgery was performed in 116 (68.6%) patients, and 49 (29.0%) patients underwent mastectomy. RCB 0 (pCR: no residual invasive tumor in the breast or lymph nodes) was detected in the surgical specimens of 90 (53.2%) patients. RCB I, II, and III were detected in 35 (20.7%), 39 (23.1%), and 5 (3%) tumors, respectively. The median residual tumor size was 9 (range, 1–55) mm. For IHC 2 + and 3 + cases, mean HER2/CEP17 ratios were 2.82 and 6.22, respectively, the difference being statistically significant (*p* < 0.0001), as calculated by a Welch two sample *t*-test. Our study included one case with highly heterogeneous immunohistochemical results displaying small foci of strong circumferential membranous HER2 staining (< 10% of tumor cells with 3 + staining), reported as 1 + according to ASCO/USCAP criteria but demonstrating HER2 amplification by subsequent CISH.

Clinicopathological characteristics of the included 169 patients are summarized in Table 1.

**Table 1:** Patients’ characteristics

**Variable**		***N* (%) total = 169**
**Age at first chemotherapy**	Median (years, range)	51.5 (23.2–77.5)
**BMI**	Median (kg/m^2^, range)	24.9 (17.7–42.2)
**Menopausal status**	Premenopausal Postmenopausal Unknown	70 (41.4%) 94 (55.6%) 5 (3%)
**Histological subtype**	Invasive ductal Invasive lobular Others	161 (95.3%) 2 (1.2%) 6 (3.5%)
Presence of DCIS^*^	Yes No	85 (50.3%) 84 (49.7%)
**Clinical tumor stage (mm)**	cT1 (≤ 20 mm) cT2 (> 20–50 mm) cT3 (> 50 mm)	60 (35.5%) 89 (52.7%) 20 (11.8%)
Grading*	G1 G2 G3 Unknown	5 (3.0%) 53 (31.4%) 110 (65.1%) 1 (0.5%)
ER/PR status*	Positive Negative	112 (66.3%) 57 (33.7%)
Ki67*	Median (range)	40.0 (5.0–90.0)
**HER2 immunohistochemistry**	1 + 2 + 3 +	1 (0.5%)^§^ 18 (10.7%) 150 (88.8%)
*HER2/CEP17* ratio*	Median (range)	5.30 (1.70–14.30)
*HER2* copy number*	Median (range)	9.35 (2.06–26.45)
**Residual tumor stage**	ypT1 (≤ 20 mm) ypT2 (> 20–50 mm) ypT3 (> 50 mm)	65 (38.5%) 11 (6.5%) 1 (0.6%)
**Lymph node stage**	ypN0 ypN1 ypN2 ypN3	143 (84.6%) 21 (12.4%) 4 (2.4%) 1 (0.6%)
**RCB classification**	0 I II III	90 (53.2%) 35 (20.7%) 39 (23.1%) 5 (3.0%)
**Lymph vessel invasion**	L0 L1	152 (89.9%) 17 (10.1%)

^*^In CNB

^§^Tumor containing < 10% tumor cells with complete, circumferential, moderate membrane staining by immunohistochemistry, *HER2*-amplification confirmed by CISH

#### *HER2/CEP17* ratio and response to neoadjuvant therapy

**Fig. 1:**
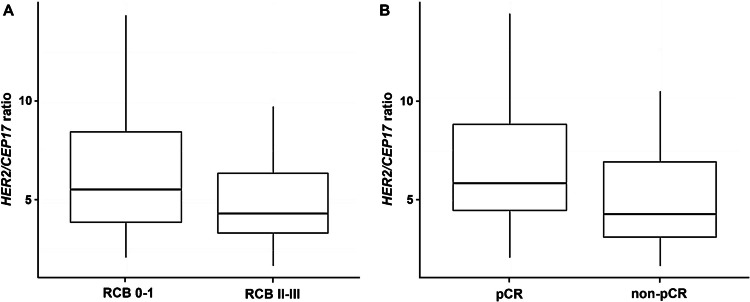
Association between median HER2/CEP17 ratio and tumor response according to residual cancer burden (**A**) and pathologic complete remission (**B**)

Median *HER2/CEP17* ratio was significantly higher (Wilcoxon, *p* = 0.005) in tumors with favorable tumor response displaying ratios of 5.54 (2.10–14.30) for RCB 0/I and 4.31 (1.70–9.70) for RCB II/III cases, respectively. Tumors achieving RCB0/pCR also displayed a significantly higher median *HER2/CEP17* ratio of 5.84 (2.10–14.30), whereas tumors with partial response had a median *HER2/CEP17* ratio of 4.29 (1.70–10.40, Wilcoxon, *p* < 0.001, Fig. 1).

Univariate logistic regression analysis indicated that the RCB 0/I category was significantly associated with lower BMI (*p* = 0.032), increased *HER2/CEP17* ratio (*p* = 0.005), higher *HER2* gene copy number (*p* = 0.015), a histological “no special type” (NST) differentiation, presence of a DCIS component (*p* = 0.014), and negative ER status (*p* = 0.003). In particular, a formerly reported *HER2/CEP17* ratio > 6 (*p* = 0.044) was also significantly associated with favorable tumor response. PR status showed no significant correlation with RCB (Table 2).

**Table 2:** Univariate analysis of factors predictive of tumor response

Variable	RCB			pCR		
Predictors	OR	95% CI	*P* value	OR	95% CI	***P*** value
BMI	1.07	1.01–1.15	0.032	0.96	0.90–1.01	0.125
Age at first chemotherapy	1.00	0.97–1.03	0.981	1.01	0.98–1.04	0.614
Menopausal status	1.11	0.55–2.23	0.781	1.19	0.64–2.20	0.590
*HER2/CEP17* ratio*	0.81	0.69–0.94	0.005	1.25	1.10–1.41	0.001
*HER2* gene copy number*	0.90	0.82–0.98	0.015	1.12	1.04–1.20	0.003
Histological subtype* NST vs others	5.21	1.19–22.81	0.028	0.12	0.01–0.99	0.048
DCIS component*	0.41	0.20–0.83	0.014	2.98	1.60–5.59	0.001
Grading* (G1/G2 vs. G3)	0.69	0.34–1.40	0.301	1.43	0.76–2.71	0.273
HR status*	2.90	1.25–6.76	0.014	0.26	0.13–0.52	0.000
ER*	1.55	1.16–2.07	0.003	0.55	0.43–0.70	0.000
PR*	1.28	0.97–1.70	0.083	0.69	0.53–0.89	0.005
Ki67*	0.99	0.98–1.01	0.514	1.00	0.99–1.02	0.598
Tumor size (cT)	1.49	0.87–2.53	0.144	0.80	0.50–1.28	0.349
Focality	1.06	0.72–1.56	0.768	1.28	0.68–2.42	0.440

^*^In pretherapeutic core needle biopsy

RCB 0/pCR was significantly associated with a higher *HER2/CEP17* ratio (*p* = 0.001), higher number of *HER2* gene copies (*p* = 0.003), presence of a DCIS component (*p* = 0.001), and negative HR status (*p* < 0.0001).

In multivariate analysis, HR status (*p* = 0.015 and *p* = 0.0001) and the presence of a DCIS component (*p* = 0.015 and *p* = 0.0006) were significantly associated with both RCB and pCR.

**Fig. 2:**
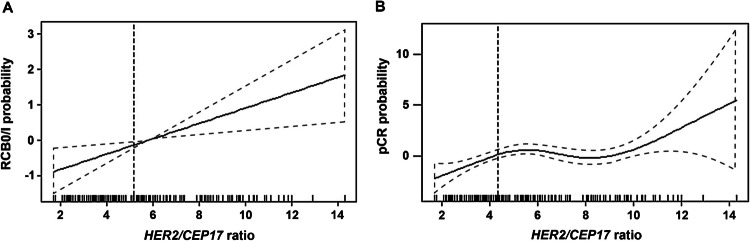
Functional relationship between the log odds and the human epidermal growth factor receptor 2/chromosome enumeration probe 17 ratio and residual cancer burden (**A**) and pathologic complete remission (**B**)

The univariate logistic regression model showed that dichotomization of the *HER2/CEP17* ratio at 6 was a significant predictor for RCB but not for pCR. The analysis indicated that for RCB, the functional relationship between the log odds and the *HER2/CEP17* ratio was almost linear (Fig. 2A). Using genetic algorithms, statistical analysis yielded an optimal cutoff *HER2/CEP17* ratio of 5.19 to predict a favorable therapy response (RCB 0/I). However, with respect to pCR, an optimal cutoff at a *HER2/CEP17* ratio of 4.37 was retrieved. For pCR, a nonlinear functional relationship between the log odds and the *HER2/CEP17* ratio was observed, with a local maximum close to 6 (Fig. 2B).

For both outcome variables, selection based on the BIC resulted in multivariate logistic regression models that included only the presence of a DCIS component and negative HR status.

### Progression-free and overall survival

Median follow-up time was 63.7 (27.5–100.9) months. Recurrence occurred in 16 (9.5%) patients, 13 (7.7%) of whom developed metastatic disease. Both RCB and pCR were strongly associated with PFS. Patients with favorable tumor response (RCB 0/I) had significantly longer PFS than those with RCB II/III (log-rank *p* = 0.03). Similarly, patients with pCR had a significantly longer PFS than those with residual tumor (log-rank *p* = 0.02).

**Fig. 3:**
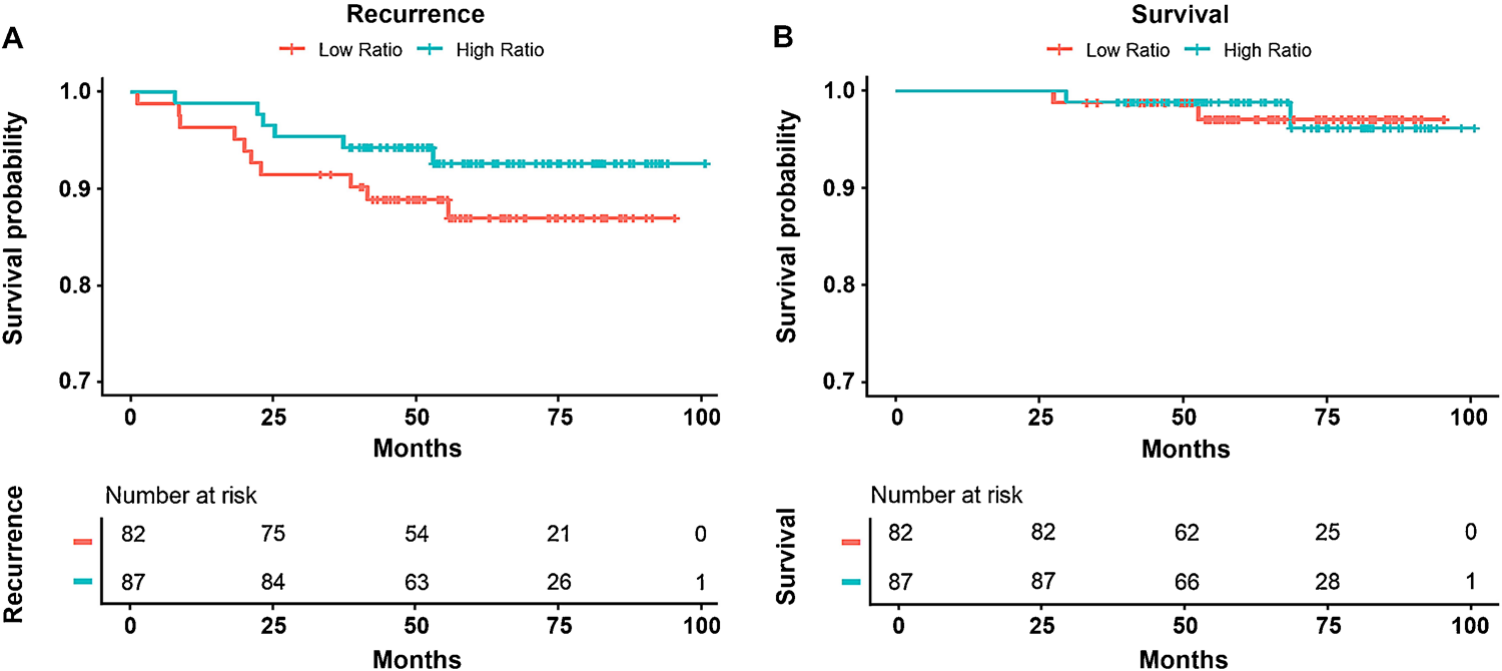
Survival curves for progression-free and overall survival using a cutoff of 5.19 for human epidermal growth factor receptor 2/chromosome enumeration probe 17 ratio

Kaplan–Meier curves for PFS and OS are depicted in Fig. 3. Cox regression models identified a significant association between *HER2/CEP17* ratio and PFS (*p* = 0.027). After dichotomizing *HER2/CEP17* ratio at the optimal cutoff points for the two primary outcome variables, the effect was no longer significant. For a cutoff value of 4.37, which was optimal with respect to the outcome of pCR, there was no significant difference between the Kaplan–Meier curves of the two groups (log-rank *p* = 0.40). At a *HER2/CEP17* ratio threshold of 5.19, which was optimal for the outcome RCB 0/I, the two groups were almost balanced and the Kaplan–Meier curves were well separated, but a total of only 16 events did not render the difference significant (log-rank *p* = 0.20). At a *HER2/CEP17* ratio threshold of 8, all events fell below the threshold in the low-ratio group, and the observed difference was the largest (log rank *p* = 0.02). However, more data would be required to define a suitable threshold for *HER2/CEP17* ratio with respect to PFS.

Within the follow-up period, four (2.4%) women died; therefore, this study did not have sufficient events to reliably estimate OS. Kaplan–Meier curves indicated a similar trend as that for PFS, but no inference could be made from the data owing to a lack of statistical power.

## Discussion

The current development of individualized risk-adapted treatment strategies for early-stage HER2-positive breast cancer aims at de-escalation and requires reliable predictive biomarkers. Our study demonstrated that *HER2/CEP17* ratio was a significant predictor for both RCB 0/I and pCR and for PFS in patients with HER2-positive breast cancer treated with neoadjuvant chemo-immunotherapy using pertuzumab and trastuzumab. With increasing threshold values for the *HER2/CEP17* ratio, the differences in therapeutic outcomes became more pronounced. A *HER2/CEP17* ratio ≥ 5.19 predicted a favorable tumor response (RCB 0/1) after dual HER2 blockade. A cutoff ≥ 4.37 was predictive for pCR, although the functional relationship between *HER2/CEP17* ratio and the log odds of pCR was nonlinear. By applying a threshold of *HER2/CEP17* ratio ≥ 8, we identified all patients without disease progression in our cohort. However, more data are required to generally define an optimal threshold for survival prediction.

Although the *HER2/CEP17* ratio is a continuous variable and defining an exact cutoff will always be relatively arbitrary, there is an urgent clinical need to identify patients with a favorable response to current neoadjuvant HER2-directed therapy regimens, eventually allowing for the de-escalation of treatment intensity. Recent trials demonstrated that a subset of HER2-amplified tumors might potentially be overtreated by the current standard of combined chemo-immunotherapy [13, 36, 37]. The phase II WSG-ADAPT-HER2 +/HR– trial involving 134 patients indicated that postoperative standard chemotherapy might safely be omitted in patients with pCR after only 12 weeks of dual HER2 blockade plus weekly paclitaxel with excellent long-term survival [11]. In the PAMELA and PHERgain trials, the HER2-enriched subtype, defined by the PAM50 assay, within a cohort of HER2-positive tumors was a strong predictor of pCR, making it feasible for patients with the HER2-enriched subtype to forgo chemotherapy [13, 37–39].

However, a low level of *HER2* amplification is often associated with failure to achieve pCR [40]. Among these tumors, the luminal-type *HER2-*amplified subtype is overrepresented [41, 42]. Phase 2 trials, such as WSG-TP-II and WSG-ADAPT-HER2 +/HR +, showed that de-escalated neoadjuvant endocrine therapy in combination with dual HER2 blockade or antibody–drug conjugates (ADCs) was a viable option in a subset of these patients, in which higher levels of HER2 protein or mRNA expression were the most significant predictors of response and outcome [43, 44].

These findings indicate that the magnitude of *HER2* amplification is likely to represent a crucial predictive factor for therapeutic response and aid in patient stratification for treatment de-escalation or escalation. Zheng et al. reported recently that tumors with a *HER2/CEP17* ratio of > 7, termed “ultra-positive,” demonstrated inferior DFS than the normal-positive group among non-metastatic HER2-positive breast cancer patients receiving trastuzumab alone [45]. However, this does not seem to be sufficiently accounted for by the current diagnostic guidelines, independent of the analytical method applied.

Novel, automated methods such as dual double in situ hybridization (D-DISH) for assessing *HER2* copy number and *HER2/CEP17* ratio are likely to speed up the development of reliable predictive factors [46]. Recently developed gene expression-based assays have been shown to provide prognostic information beyond standard clinicopathological parameters with reliable analytical validity [47]. However, their costs significantly exceed those of ISH analysis and are currently not endorsed by regulatory authorities. In contrast, ISH can be easily performed in most pathology laboratories, even in low-resource settings. Other PCR-based methods may also fulfill this task efficiently [48]. Furthermore, advances in digital pathology and automation bear great potential to facilitate rapid analysis of FISH/CISH images and are likely to improve the accuracy of analyses [49].

The potential limitations of our study include its retrospective design and single-institution setting. Nevertheless, this is sufficiently counterbalanced by a homogenous patient cohort with well-described clinicopathological characteristics and consensual pathology reporting.

Our results suggest that in *HER2*-amplified early breast cancer, the exact *HER2/CEP17* ratio is an independent, relevant tumor parameter for stratifying patients for neoadjuvant therapy de-escalation. Our results correspond well with earlier studies that identified the *HER2/CEP17* ratio as an important predictive factor, independent of HER2 immunohistochemistry, in advanced HER2-amplified breast cancer [29]. Moreover, we could also demonstrate a linear relationship between the *HER2/CEP17* ratio and tumor response by RCB. Future clinical trials are warranted to investigate whether tumors with a high *HER2/CEP17* ratio (of ≥ 5.19) benefit from therapy de-escalation without chemotherapy. On the other hand, luminal HER2-positive tumors with a lower *HER2/CEP17* ratio may be better served by alternative, less selective anti-HER2 strategies, such as ADCs or concomitant chemotherapy. Our results may provide a basis for revisiting the current uniform non-stratified definition of HER2 positivity. The development of novel therapeutic regimens should go further hand-in-hand with the validation of predictive biomarkers at the individual patient level, challenging current standards to accelerate progress in breast cancer management.

## Data Availability

The datasets generated and analyzed during the current study are available from the corresponding author on reasonable request.

## Abbreviations

HER2: Human epidermal growth factor receptor 2
HER3: Human epidermal growth factor receptor 3
CEP17: Chromosome enumeration probe 17
RCB: Residual cancer burden
pCR: Pathologic complete remission
PFS: Progression-free survival
OS: Overall survival
CISH: Chromogenic in situ hybridization
FISH: Fluorescent in situ hybridization
DCIS: Ductal carcinoma in situ
*ERBB2*: Erb-B2 receptor tyrosine kinase 2 gene
BIC: Bayesian Information Criterion
